# Mathematical and computational modeling of biosystems at different levels of organization

**DOI:** 10.18699/vjgb-26-21

**Published:** 2026-05

**Authors:** S.A. Lashin, R.A. Ivanov, Y.G. Matushkin

**Affiliations:** Institute of Cytology and Genetics of the Siberian Branch of the Russian Academy of Sciences, Novosibirsk, Russia; Institute of Cytology and Genetics of the Siberian Branch of the Russian Academy of Sciences, Novosibirsk, Russia; Institute of Cytology and Genetics of the Siberian Branch of the Russian Academy of Sciences, Novosibirsk, Russia

**Keywords:** mathematical modeling, biological systems, multiscale models, computational biology, математическое моделирование, биологические системы, многомасштабные модели, компьютерная биология

## Abstract

Modern biology increasingly relies on mathematical and computational modeling to describe complex hierarchically organized biological systems. This review considers models that cover the main levels of biological organization, from the molecular-genetic and cellular levels to tissue/organ, organismal, population and ecological ones. The aim of the work is to systematize the key modeling approaches at each of these levels, to analyze their capabilities and limitations, and to discuss strategies for constructing multiscale and hybrid models that consistently link processes operating at different spatial and temporal scales. We survey classical deterministic and stochastic models based on ordinary and partial differential equations, logical and graph-based models of regulatory networks, cellular automata, agent-based models, as well as flux-balance approaches. Typical examples are given for the modeling of gene regulatory and metabolic networks, chemotaxis, tissue and organ growth, population dynamics and genetic structure, and ecosystem functioning. Special attention is paid to comparing approaches with respect to the scale of description, complexity of modeled processes, data requirements, computational cost and interpretability of results. The analysis shows that hybrid and multiscale models provide an adequate framework to account for nonlinearity, stochasticity and structural heterogeneity of biosystems, but require substantial computational resources and careful data-driven calibration. Methodological and technological trends are outlined, including the development of specialized platforms and model repositories, standards for model representation and tools for reuse of model components.

## Abstract (ru)

Современная биология все в большей степени опирается на математическое и компьютерное моделирование для описания сложных иерархически организованных биосистем. В данном обзоре рассматриваются математические модели, охватывающие основные уровни биологической организации – от молекулярно-генетического и клеточного до тканевого/органного, организменного, популяционного и экологического. Цель работы состоит в систематизации ключевых подходов к моделированию на каждом из этих уровней, анализе их возможностей и ограничений, а также в обсуждении стратегий построения многомасштабных и гибридных моделей, связывающих воедино процессы разных пространственно-временных масштабов. Рассматриваются классические детерминированные и стохастические модели на основе дифференциальных уравнений в частных производных, логические и графовые модели регуляторных сетей, клеточные автоматы, агентно-ориентированные и индивидуально-ориентированные модели и подходы, базирующиеся на балансе потоков. Приводятся типичные примеры моделирования молекулярно-генетических сетей, метаболизма и хемотаксиса, роста тканей и органов, динамики популяций и генетической структуры, а также функционирования экосистем. Особое внимание уделяется сопоставлению подходов по критериям масштабов описания, сложности моделируемых процессов, доступности исходных данных, вычислительной трудоемкости и интерпретируемости результатов. Обзор обобщает отечественный и зарубежный опыт, подчеркивая вклад российских и, в частности, новосибирских коллективов в развитие гибридных методов моделирования, построения многомасштабных моделей и реализации программных платформ для системной биологии. В результате проведенного анализа показано, что гибридные и многомасштабные модели позволяют наиболее полно учесть нелинейность, стохастичность и структурную неоднородность биологических систем, но требуют значительных вычислительных ресурсов и тщательной калибровки по данным. Отмечаются методические и программно-технологические тенденции, включая развитие специализированных платформ и репозиториев моделей, средств стандартизации описания и повторного использования модельных компонентов.

## Introduction

Biological systems are complex, hierarchically organized systems where differences in the characteristic sizes of objects span up to ten orders of magnitude, and differences in the characteristic timescales of processes span up to 18 orders of magnitude (Riznichenko, 2003; Shumnyi et al., 2006).

Highlighting the levels of biological organization that are most developed in terms of mathematical description, we note the following: • Molecular-genetic; • Cellular; • Tissue/organ; • Organismal; • Population; • Ecological/biocenotic

Each of these levels is characterized by specific objects and processes and, accordingly, requires its own mathematical and computer models for description. It should also be noted that concepts such as genotype and phenotype, being among the most frequently used terms in biology, can be described from the perspective of mathematical modeling in completely different ways. Depending on this description, they may formally belong to different levels of biological organization. Depending on the context of the situation under consideration, these allelic variants can be described as symbolic sequences, numbers, etc. The diversity of ways to describe phenotypic traits of an organism is even broader – these can be discrete traits (e. g., eye or hair color, flower color) or continuous traits (e. g., height or weight). Traits can be the result of measurements conducted using both simple instruments like rulers or scales, and complex physical and/or biochemical instruments (e. g., morphophysiological traits).

The aim of this review is to systematize the main mathematical and computer methods for modeling biological systems at various levels of organization, analyze their capabilities and limitations, and consider approaches to constructing multiscale and hybrid models that combine several levels within a unified conceptual and computational framework.

## Molecular-genetic level of organization

The Molecular-Genetic Control System (MGCS) of a cell is the set of its irregular polymers (DNA, RNA, and proteins), as well as molecular subsystems performing various biochemical processes on these irregular polymers (Ratner et al., 1985): Synthesis; • Transformation; • Decay; • Transport, etc.

Among the methodological approaches for studying the MGCS, three main ones can be noted: 1. Structural-functional approach – focuses on the material properties of macromolecules and the MGCS in relation to their function. It studies structural, physicochemical, and energy patterns of macromolecule structure, thermodynamics and kinetics of processes, etc. 2. Information-cybernetic approach – focuses on identifying the principles of organization and control of the MGCS, abstracting from their structural features. It studies self-reproduction, information processes, coding, memory, reliability, regulation systems, etc. 3. Evolutionary approach – identifies the paths of origin and evolution of the MGCS as a whole, various subsystems and fractions of macromolecules, as well as evolution factors, types of evolutionary dynamics, etc. (Ratner et al., 1985).

A particular case of the MGCS is the so-called gene networks – groups of coordinately functioning genes interacting with each other both through their primary products (RNA and proteins) and through various metabolites and other secondary products of gene network functioning (Kolchanov et al., 2013).

Models of biological systems at the molecular-genetic level include simple ODEs (Ordinary Differential Equations) and systems of ODEs consisting of several equations, discrete models built using various formalisms (Boolean networks, Petri nets, cellular automata, etc.), discrete-continuous models, as well as various computer simulation and agent-based models.

The simplest MGCS models were built based on chemical kinetics equations of the form (Eq. (1)), representing various ways of presenting the kinetic law of mass action and the Generalized Chemical Kinetic Modeling Method (GCKMM) proposed by Vitaly Likhoshvai (Likhoshvai et al., 2000):

**Eq. 1.**
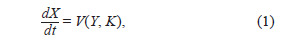
Eq. 1.

where X – vector (list) of controlled variables, Y – vector (list) of controlling variables, K – list of parameters.

The inclusion of the same variables in both lists is allowed, but, in general, the lists X and Y do not coincide and may not intersect at all. Variables usually represent concentrations of substances or probabilities of realizing selected states of substances. Variables from the list Y not included in the list X are parameters for the current elementary model. The functional V describes the law of rates of change of concentrations of substances from the list X.

The kinetic law of mass action is derived based on collision theory. Let there be a biochemical reaction:

**Eq. 2.**
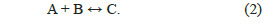
Eq. 2.

Then the rate VA + B → C of formation of complex C at current time t is equal to:

**Eq. 3.**
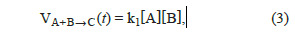
Eq. 3.

and the rate VC → A + B of decay of complex C into components A and B at current time t is equal to:

**Eq. 4.**
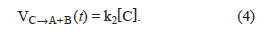
Eq. 4.

If there is a specific biochemical reaction scheme of the form (Eq. (2)), then the instantaneous rate of change of concentration of any substance equals to the sum of local rates of change of concentration of this substance in each reaction in which this substance participates (Kazantsev et al., 2009; Akberdin et al., 2013). This simple rule allows easily writing down the final system of differential equations describing the target biochemical scheme, using only first and second-order polynomials as the right-hand sides of the system equations (Eq. (1)). The theoretical basis for it is the Korzukhin theorem, which is crucial for modeling chemical kinetics: “For any set of non-negative curves defined on a finite time interval, and any given accuracy, there exists such (perhaps not the one) biochemical scheme, composed only of bimolecular and monomolecular reactions, that the mathematical model built according to this biochemical scheme approximates the given set of curves with the given accuracy” (Zhabotinskii, 1974).

The most important element in constructing more complex MGCS models using GCKMM (Likhoshvai et al., 2000) is the rule of summation of local rates of biochemical reactions: the total rate of change of component concentrations in the system is the sum of the rates of change of concentration of this component in all elementary processes (see Supplementary Materials, Table S1 for details)^1.^

Supplementary Materials are available in the online version of the paper: https://vavilov.elpub.ru/jour/manager/files/Suppl_Lashin_Engl_30_3.pdf


Logical approaches for modeling MGCS, introduced back in the 60s of the 20th century, are based on applying logic and discrete mathematics terms to describe molecular-genetic mechanisms (Kauffman, 1969). Let us give an example of describing gene network operation in terms of Boolean logic. A gene network is represented as a Boolean network – a directed graph where vertices represent gene states, and edges represent regulatory events, i. e., the action of genes on other genes (Ratushnyi et al., 2005; Tran, 2016; Barbuti et al., 2020).

The current state of the gene network is described by a list of expressed (working) and non-expressed (non-working) genes at discrete moments in time. It is assumed that genes can be in only two states: “expressed” (true,1), or “not expressed” (false,0). The state of the gene network, thus, represents a Boolean vector:

**Eq. 5.**
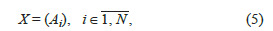
Eq. 5.

where Ai is the state of the i-th gene. The change of network states is described using a Boolean vector-function f :

**Eq. 6.**
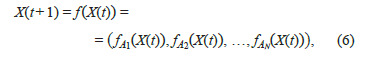
Eq. 6.

where fAi:{0,1}N → {0,1} – describes the impact of expression of all genes on the expression of the i-th gene. An example of a simple Boolean gene network is shown in the Figure

**Fig. 1.**
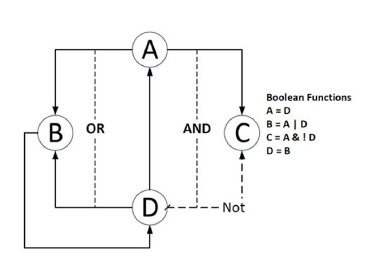
Figure.

It should be noted that the approaches mentioned above have limited applicability in quantitative forecasting due to their simplification. Therefore, at present, the most frequently used approaches for describing MGCS are various hybrid methods combining continuous, discrete, and stochastic modeling methods. One of the first such methods is the generalized threshold modeling method developed in Novosibirsk by Rustem Churaev back in the 70s of the 20th century (Churaev, Ratner, 1972; Churaev, 2005). This method combines approaches of automata theory and linear ODEs. The core idea lies in dividing the system’s phase space into regions, within which a system behavior is described by linear ODEs, while transition conditions between regions are described by Boolean functions. The solutions of linear systems are “stitched” at zone boundaries. Another example of hybrid approaches is a rule-based modeling, where the model is defined indirectly via a specific set of rules (Blinov et al., 2004; Harris et al., 2016). Further development of GCKMM and numerical modeling methods of MGCS based on it at the Institute of Cytology and Genetics was also carried out within the paradigm of hybrid approaches (Kazantsev et al., 2009, 2018).

## Cellular level of organization

Mathematical modeling at the cellular level of organization considers biological processes such as transport of substances and energy from the environment into the cell and back, their metabolism, cell movement in space, and their division. Regarding modeling metabolic processes, the cellular level of organization is directly linked to the molecular-genetic level discussed in the previous section. The term “Electronic Cell”, which appeared in the late 20th–early 21st century (Tomita et al., 1999; Tomita, 2001; Ishii et al., 2004; Price et al., 2004; Karr et al., 2012; Akberdin et al., 2013), implies modeling the cell primarily at the molecular-genetic level of biological organization

Nevertheless, unlike the models discussed in the previous section, where the MGCS themselves, their states, functioning modes, etc., are of interest, cell models can focus on other events and processes, considering the MGCS models included in them as “handy tools”. For example, Japanese systems biologist and bioinformatician Masaru Tomita, the leader of the E-CELL project – one of the first successful projects modeling an electronic bacterial cell – highlights among cellular level organization processes that can be modeled using MGCS processes: substance transport across the membrane, cell cycle and cell division, as well as the development of pathological states (e. g., for human erythrocyte cells) (Tomita et al., 1999; Tomita, 2001; Hucka et al., 2003). Programs developed within the E-CELL project use a hybrid approach to modeling cell viability; in particular, version E-CELL 3 uses ODE systems, stochastic modeling using the Gillespie algorithm, as well as special algorithms for “stitching” solutions.

Another important cellular process for modeling which the MGCS models can also be used is chemotaxis – cell movement. Bacterial chemotaxis represents one of the simplest and wellstudied examples of microbial behavior. It allows swimming bacterial cells to follow chemicals in the environment along a concentration gradient. Molecular mechanisms of chemotaxis in the model bacterium E. coli have been studied in great detail over the last 50 years, using a wide spectrum of experimental, mainly biophysical, methods (Berg, Purcell, 1977; Vladimirov, Sourjik, 2009; Kaizu et al., 2014). The accumulation of experimental data in this area led to the creation of several chemotaxis models. For example, the model published by the group (Bray et al., 1993) is a hybrid block-modular model where the molecular-genetic component is described using chemical kinetics equations, and the physical component – rotation of special motor proteins – using a finite automaton. A schematic diagram of this model is provided in Figure S1. The model reproduces the behavior of over 30 mutants, indicating its high quality. The model was developed and implemented as the BCT (BacterialChemoTaxis) software package (Bray, Bourret, 1995), which subsequently allowed describing over 60 mutants (Bray et al., 2007). Additionally, agent-based models combining this approach with stochastic modeling of MGCS were used to study chemotaxis (AgentCell (Emonet et al., 2005)); hybrid modeling combining ODE blocks and stochastic modeling, as well as mean-field approximation for the Monod–Wyman–Changeux model The Monod–Wyman–Changeux model describes the regulation of enzymatic activity in a protein composed of identical subunits through allosteric structural changes (Monod et al., 1965).(RapidCell (Vladimirov et al., 2008), see also Fig. S2).

Molecular mechanisms of movement in more complex eukaryotic cells differ fundamentally from those in bacteria. Nevertheless, the methodological repertoire for mathematical modeling of these processes is generally the same. For example, a mathematical model of hair cell regulation – receptors of the auditory system and vestibular apparatus of animals and humans, describing ion transport, membrane potential, and cell movement (O’Beirne, Patuzzi, 2007), was implemented based on the Boltzmann equation.

Despite the significant background in modeling biological systems at the cellular level of organization, in most studies currently being conducted, single-cell models are used as auxiliary tools. This applies to levels of biological organization considering models of cell ensembles – tissue, organismal, and population levels.

## Tissue/organ level of organization

Biological tissue is a system of cells similar in origin, structure, and functions performed in the organism, as well as intercellular substances and structures – products of their vitality (Gilyarov et al., 1986). Animal tissues are divided into four main types: connective, muscle, nervous, and epithelial. Plant tissues are divided into simple, consisting of cells of one type (e. g., collenchyma), and complex, consisting of different types (e. g., epidermis). Depending on the classification, two or three types of plant tissues are distinguished. In the first case, tissues are subdivided into meristematic (actively dividing cells, e. g., in roots or stem tips) and permanent (having lost the ability to divide). In the second case, tissues are divided into tissue systems: epidermis, mechanical tissue, and conducting tissue (Gilyarov et al., 1986). A combination of various interacting tissues forms organs.

Given such diversity of properties and functions of biological tissues and organs, the repertoire of mathematical and computer models representing this level of biological organization is extremely large. The methodological arsenal for constructing such models is broad and includes both classical modeling methods using systems of ODEs and partial differential equations (more often used for modeling biological tissues), and modern hybrid modeling methods including agent-based approaches, etc.

Mathematical and computer modeling of biological tissues and organs arouses the greatest interest among medics and biologists in the following contexts: (1) modeling development, i. e. morphogenesis, embryogenesis, etc.; (2) modeling pathologies (diseases) and strategies for their correction. In some cases, the same model can be considered in both contexts simultaneously, for example, a cancer tumor development model allowing numerical investigation of various treatment strategies.

The classical model of morphogenesis theory is the “reaction- diffusion” model proposed in the mid-20th century by Alan Turing (Turing, 1952):

**Eq. 7.**
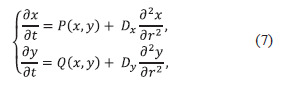
Eq. 7.

where r – spatial coordinate, Dx∂2x/∂r2 and Dy∂2y/∂r2 describe diffusion of substances x and y along this coordinate. Diffusion of non-linearly linked components x and y in this system leads not to averaging, but to a distribution periodic in time and non-uniform in space (Riznichenko, 2003)

Modifications of the “reaction-diffusion” model have been actively studied and applied to a wide range of biological tasks, including modeling tissues and organs. In particular, a mathematical model implemented as system (8) was used to study animal skin coloration patterns. Depending on model parameters, which correspond to different combinations of morphogens (substances affecting individual organism development), coloration patterns vary within a wide range (Fig. S3).

**Eq. 8.**
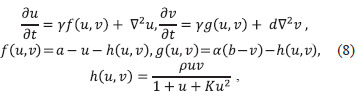
Eq. 8.

where f, g, h – functions describing reaction kinetics; a, b, α, ρ and K – positive parameters describing reaction kinetics, representing ratios of kinetic constants (detailed derivation of these parameters is provided in (Murray, 2003)); d – ratio of diffusion coefficients; γ determines the region size

Another approach to modeling biological systems at the tissue and organ level of organization was proposed by Vitaly Likhoshvai and later developed in works under the supervision of Victoria Mironova on modeling plant tissues (Likhoshvai et al., 2007; Fadeev et al., 2008; Mironova et al., 2010, 2012; Novoselova et al., 2013; Pasternak et al., 2019). Its essence lies in describing plant tissue as a system of ODEs of sufficiently large dimension – from hundreds to several thousand equations. Here, a separate tissue cell corresponds to several equations; in most works – just four equations describing the dynamics of substances of interest to the researcher: proteins and hormones. Cells are considered as systems with complete mixing. Redistribution of substances between cells via diffusion and active transport processes is described by substance transport equations. An example of such equations for the hormone auxin is given in the following fragment of system (9) from the article (Likhoshvai et al., 2007) (model scheme is shown below in Fig. S4).

**Eq. 9.**
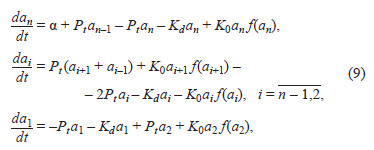
Eq. 9.

where ai – concentration of hormone auxin in the i-th cell of the plant root (the root itself is considered in this model as a one-dimensional array of cells, see also Fig. S4a); parameter α describes constant auxin inflow into the system; Pt – auxin diffusion between cells; Kd – auxin degradation parameter in the cell; function f (ai) describes active directed transport of auxin across the membrane via special transporter proteins (Eq. (10)):

**Eq. 10.**
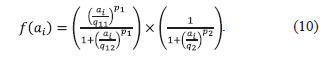
Eq. 10.

In this function, in turn, q11 – activation threshold constant for auxin-dependent transport; q12 – saturation threshold constant for auxin-dependent transport; q2 – inhibition threshold constant for auxin-dependent transport; p1 and p2 – nonlinearity coefficients of activation and inhibition mechanisms, respectively. In the aforementioned series of works on modeling plant organs and tissues, both one-dimensional (1D) and two-dimensional (2D) models were considered (Fig. S4). Although these models represent systems of ODEs (often of large dimension), ideologically they are close to cellular automaton models, which are also frequently used for modeling biological systems at tissue and organ levels of organization.

Thus, modeling using cellular automata was used to study ontogenesis processes (Markus et al., 1999; Akberdin et al., 2007; Vitvitsky, 2014; Pałubicki et al., 2019) and vegetation (Komarov et al., 2003; Colasanti et al., 2007) of plants. This approach is also actively used for modeling tissues and organs of animals and humans, in particular, pathological states such as oncological diseases (Gevertz, Torquato, 2006; Szabó, Merks, 2013; Brüningk et al., 2019; Salguero et al., 2019), immune (Bezzi et al., 1997), infectious (Slimi et al., 2009) and others (Talaminos-Barroso et al., 2020).

In most of the works listed above, as well as in many others not included in this review, the cellular automaton modeling methodology is used in combination with other approaches, in particular, agent-based modeling and rule-based modeling (Fig. S5), mentioned earlier in section “Molecular-genetic level of organization”. Such hybrid models, used primarily for modeling tissues and organs, received the name Cellular Potts Models (Glazier, Graner, 1993; Marée et al., 2007; Voss- Böhme, 2012).

Further development of methods for modeling biological tissues and organs led to the emergence of software packages, libraries, and platforms adapting Potts models and agent-based modeling for solving specific content-related tasks in biology. We note such software packages as CellSys (Hoehme, Drasdo, 2010), EPISIM (Sütterlin et al., 2013, 2017), CompuCell3D (Swat et al., 2012), and others. We separately note projects on modeling whole organs, for example, the platform for modeling the human liver VirtualLiver (Holzhütter et al., 2012; Drasdo et al., 2014). The development of methods for modeling biological tissues and organs prepared the background for the emergence of computer models of whole multicellular organisms, which will be discussed in the next section.

## Organismal level of organization

The history of mathematical and computer modeling of individual organism functioning dates back to the 60s of the 20th century. The first and simplest tree model was presented by Igor Poletaev and his students in 1965 (Poletaev, 1965, 1966). Despite its simplicity, the model, based on physical principles, answered the question “why does a tree not grow infinitely in height?” Subsequently, more complex model variants were built and investigated based on the simplest model (Karev, Skomorovsky, 1999; Kolobov, Frisman, 2008). The real flourishing of detailed models of individual multicellular organisms occurred in the last 10–15 years. This was largely due to the colossal progress achieved in computer performance, namely – processing speed, RAM volume, and storage device capacities. It became possible to create realistic models of living organisms. The term “Digital Twins of Biological Organisms” appeared (Barnabas, Raj, 2020; Tellechea-Luzardo et al., 2020; Miehe et al., 2021), etc.

The methodological arsenal used for modeling at the organismal level of biological organization naturally includes all approaches discussed in previous sections. Depending on the goals and tasks that authors of “digital organisms” set for themselves, some levels of biological organization, as well as corresponding biological processes, may be described in the model in more detail, and others – less. For example, in the well-known OpenWorm project, dedicated to creating a computer model of the worm Caenorhabditis elegans (Szigeti et al., 2014; Sarma et al., 2018; Palyanov, 2019), main attention is paid to modeling movement biomechanics and biophysics of neuroimpulse transmission. Whereas, for example, the Digital Salmon project (Omholt et al., 2013) is more focused on salmon metabolism and ontogenesis processes.

It should be noted that the work on creating computer models of whole organisms – “digital twins” – is currently being carried out by large scientific teams, and often by consortia consisting of many teams. As a rule, within these works, entire software packages and even software platforms are developed, which contribute to the development of the methodological base of mathematical and systems biology

## Population level of organization

Biological population – a collection of individuals of one species possessing a common gene pool and occupying a certain territory (Gilyarov et al., 1986). The history of mathematical biology is primarily linked to modeling biological populations. Starting with Leonardo Fibonacci, who in his arithmetic book “Liber Abaci” proposed a model of rabbit population size change over time (the model solution – the famous Fibonacci numbers), continuing with Thomas Robert Malthus’s work “An Essay on the Principle of Population” (Malthus, 1978) and Pierre François Verhulst’s “Notice on the Law that Population Follows in its Growth” (Verhulst, 1838), it is precisely population dynamics modeling that becomes the driving force of mathematical biology development. The models described in the mentioned works are, of course, very simple (see Eq. (11) and (12)), but they have served as a foundation for more complex models not only in biology but also in other fields of science – physics, chemistry, etc. Below is the Malthus equation:

**Eq. 11.**
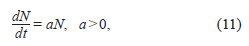
Eq. 11.

where N – population size, a – population growth rate coefficient.

The Verhulst equation looks as follows:

**Eq. 12.**
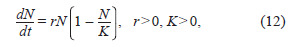
Eq. 12.

where r – population growth rate coefficient, K – maximum population size.

Models of interacting populations were first proposed by Alfred J. Lotka (Lotka, 1909, 1920, 1925) and Vito Volterra (Volterra, 1928, 1976) and subsequently received the names “Lotka–Volterra models” or “predator–prey models”:

**Eq. 13.**
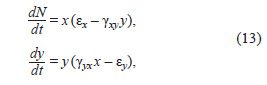
Eq. 13.

where x – number of prey, y – number of predators; coefficients: εx – natural prey growth, εy – natural predator decline (in absence of prey), γxy – impact of predators on prey numbers, γyx – impact of prey on predator numbers

The generalized Volterra model (Eq. (14)) allows considering other types of interactions between two populations besides “predator–prey” relationships

**Eq. 14.**
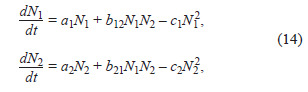
Eq. 14.

where N1, N2 – size (density) of corresponding population, a1, a2 – growth rate of corresponding population, c1, c2 – mortality coefficient of corresponding population, b12, b21 – coefficients of population influence on each other. Depending on the values of parameters b12 and b21, the type of interaction between populations is determined (see the Table).

**Table 1.**
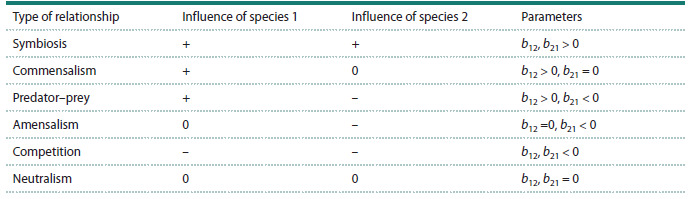
Types of interactions between populations considered in the generalized Volterra model (Eq. (14)), according to: (Odum, 1975)

Functioning modes realized in models (13, 14) boil down to two types – stationary states of the system and oscillating modes (Riznichenko, 2002, 2017). When several (more than two) populations interact with each other, the richness of dynamic modes of models increases sharply. For example, in the “predator–two prey” system, the possibility of existence of chaotic modes was shown (Aponina et al., 1982).

In the book by Alexander Bazykin “Nonlinear Dynamics of Interacting Populations”, an exhaustive analysis of models of three interacting populations of the generalized Volterra model type is conducted (Bazykin, 2003). Discrete analogs of the continuous population dynamics models discussed above – Moran and Ricker models – consider population size as a discrete quantity changing at certain discrete moments in time, which corresponds to experimental data on census of real populations. If we assume that population size at time t (Nt, t = 0, 1, 2, …) depends on sizes at some preceding moments in time, then to describe population dynamics one can apply the apparatus of recurrent or difference equations (mappings):

**Eq. 15.**
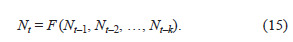
Eq. 15.

The solution of this equation is a sequence of values Nt satisfying equation (15) at each t. The Moran and Ricker model (Eq. (16)) was proposed to describe population dynamics of insects (Moran, 1950) and fish (Ricker, 1958):

**Eq. 16.**
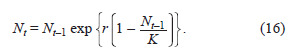
Eq. 16.

Interestingly, even in such a simple model, very different functioning modes are found (Fig. S6).

Another large direction in population modeling – population genetics modeling – was laid by classics of mathematical biology: Ronald Fisher, John Haldane, Sewall Wright, and others (Haldane, 1924, 1926, 1990; Fisher, 1930; Wright, 1931, 1949). Unlike population dynamics models describing changes in population sizes, population genetics models focus on describing changes in allele frequencies (gene variants) in populations. The mathematical apparatus used in classical population genetics models largely resembles that used in population dynamics models – these are either recurrent equations or ODEs. As an example, below is a model of a Mendelian asexual diploid panmictic population with one diallelic locus (a gene having only two states – A1 or A2):

**Eq. 17.**
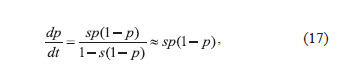
Eq. 17.

where p – frequency of allele A1 in the population (then frequency of allele A2, q = 1 – p), gamete fitness is defined as w1, w2, and their difference: s = w1 – w2. Equation (17) describes the change in allele frequencies over time

Another class of models – matrix models of population structure dynamics with complex age-sex structure or populations including individuals of one species with differing physiological or biological characteristics, first proposed by Patrick H. Leslie (Leslie, 1945, 1948), were thoroughly investigated in studies (Gimelfarb et al., 1974; Logofet, Belova, 2008). The model is represented by an equation of the form:

**Eq. 18.**
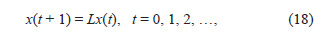
Eq. 18.

where column-vector x(t) = [x1(t), x2(t), …, xn(t)]T describes population structure (numbers of separate groups of individuals), and matrix L, also called the Leslie matrix, has the form:

**Formula:**
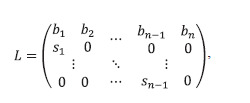
Formula

where bi – birth rate coefficients, si – survival coefficients.

Until recently, due to the lack of large-scale genomic data, such models were built primarily based on certain biologically meaningful assumptions, which allowed conducting theoretical research in this area only at a qualitative level. The development of sequencing methods and the subsequent emergence of large volumes of experimental data led to the appearance of computer models of population-genetic processes taking these data into account at a quantitative level. Simulation modeling of genetic sequence evolution received the name “coalescence simulation”.

Methodologically, coalescence modeling represents a variety of stochastic modeling using various approaches (Monte Carlo methods, Markov chains, etc.) (Salem et al., 2005). Most works in this area are based on the development and modification of classical population genetics models, such as the Wright–Fisher model (Hudson, 2002), island model (Wakeley, 2001), and others. A scheme of sequential complication of the population-genetic model by adding additional biological parameters to it was proposed (Schaffner et al., 2005) together with an algorithm for verifying values of these parameters. Currently, a number of software packages have been created for such modeling: SIMCOAL 2.0 (Laval, Excoffier, 2004), GENOME (Liang et al., 2007), Migrate-n (Beerli, Palczewski, 2010), CoaSim (Mailund et al., 2005), and others. Biologically significant results were obtained using these packages. In work (Bataillon et al., 2006), an assessment of the effective population size of Iceland, recombination rate, and a number of other population parameters was conducted. Testing new methods of analyzing genetic associations with human diseases using computer modeling is provided in (Guan et al., 2009). Statistical assessment of alternative human evolution scenarios using modeling was conducted in study (Fagundes et al., 2007).

## Ecological/biocenotic level of organization

Ecology (from Ancient Greek oikos – “house” and logos – “study”), according to Ernst Haeckel – is the science of relationships of organisms and their populations with each other and with the habitat (Haeckel, 1866). Ecology studies biocenoses and ecosystems as a result of interdependent evolution of organisms (biota) and biocenological environment, taking into account activities of populations carried out at different trophic levels, determining the power of energy and substance flows in ecosystems and the general circulation of substances, as well as autoregulation of ecosystems and their role in the planet’s biosphere (Bykov, 1983). Currently, the functioning of the planet’s ecosystems depends on social factors and anthropogenic influences. In any limited space, usually many species inhabit, between which constant and complex relationships have been established. In other words, various types of organisms existing in a certain space with a complex of physicochemical conditions form a complex system, more or less persistently preserved in nature. In ecology, they are called ecosystems (Tansley, 1935) or biogeocenoses (Sukachev, 1972).

The term “ecological modeling” includes consideration of both models of interaction of individual organisms with the environment (autecology), interaction of population with the environment (demecology), and whole communities or biocenoses (synecology). Ecological models are based, first of all, on describing the transfer of substance, energy, and information between different parts of the ecosystem. Main attention is paid to how these parts interact, how they are connected to each other and influence each other, including the physical environment.

Dimensional units used in ecological modeling are usually the amount of energy or matter moving through the system. This is one of the main differences of ecological models from population ones, where measurement units are usually population size (Jørgensen, 2009)

The methodological arsenal of ecological modeling largely repeats methods used for describing molecular-genetic, cellular, organismal, and population levels discussed in previous sections of this work. In particular, ecological modeling uses the flux balance analysis method (FBA) (Allen, Gillooly, 2009; Orth et al., 2010). The main idea of the method lies in describing substance conversion flows as a linear programming task with constraints, which gives opportunities to estimate synthesis and degradation rates of these substances. An example of a schematic image of a model based on flux balance principles is provided in Figure S7.

ODE methods (Tskhai et al., 2001; Owolabi, Patidar, 2016; Lavaud et al., 2020) and systems of PDEs (Holmes et al., 1994; Tskhai et al., 2001), cellular automata (Gómez Esteban, Rodríguez-Patón, 2011), stochastic modeling (Kutalik et al., 2005; Phillips et al., 2006; Khatri et al., 2012), graph analysis (Fath et al., 2007), and other methods are also actively used in ecological modeling.

Ecological modeling is also one of the directions in which multiscale and multilevel/multilayer modeling received the strongest impulse for development (Grimm et al., 2005, 2010; Grimm, Berger, 2016).

A particular case of multiscale models are agent-based models, which in ecological modeling are traditionally called individual-based models. Being essentially simulation models, they cover a significant spectrum of ecosystem functioning questions both at the level of individual organisms and their populations and communities (Kreft et al., 1998; Doebeli, Dieckmann, 2003, 2004; Hellweger et al., 2016; Widder et al., 2016). In the individual-based paradigm, modern software packages have been developed, in particular, for modeling bacterial communities. For example, the simulator program BacSim (Kreft et al., 1998, 2001), as well as the software package developed in work (Xavier et al., 2005), describe such bacterial life processes as substrate uptake (transport), metabolism, cell division, and cell death, highlighting a separate cell as an object, considering communities as ensembles of such objects. They are oriented, first of all, towards studying bacterial communities in the form of biofilms. The program Micro-Gen Bacteria Simulator models the life cycle of a growing bacterial culture and its interaction with various molecules, for example, antibiotics (Murphy, Walshe, 2011). In these programs, ecological, metabolic, and population components are described in detail, but description of genetic processes and inheritance is absent.

## Conclusion

Modern methods of modeling biological systems at different hierarchical levels of organization are based on both traditional approaches (differential, algebraic, and stochastic equations, graph theory, cellular automata, etc.) and hybrid techniques combining object-oriented and agent-based (individualbased) approaches with the traditional ones mentioned above. Although collectively they cover practically all aspects of ecological (Jørgensen et al., 2009) and evolutionary (de Jong, 2002; Ferrer et al., 2008) processes, the combination of ecological and evolutionary components within one model still remains quite rare

Comparing traditional approaches to building mathematical models with simulation modeling, we can see that in both cases certain limitations exist, significant for such a modeling object as a complexly organized biological system, such as, for example, a microbial community. In the first case, the static structure of the model acts as a limitation: the number of equations, variables, and model parameters does not change during the calculation. In the case of simulation modeling, the problem of model structure staticity is solved, as simulation models can contain a variable number of objects (e. g., individuals). However, simulation models of evolution and population dynamics are very demanding on RAM size and also have high computational complexity.

Based on the above, development of modeling methods for complex hierarchically organized biological systems, taking into account multiscale processes occurring in these systems, as well as taking into account their evolution – is an important task of modern mathematical biology. No less important is the task of developing software packages allowing effective solving of content-related biology tasks using mathematical and computer modeling.

It should be separately emphasized that the role of mathematical and computer models in biotechnology will only grow. Modeling biotechnological processes – from kinetics of enzymatic reactions and bioreactor operation to rational design of metabolic pathways and optimization of producer strains – allows not only reducing the cost and duration of experimental search but also purposefully forming the solution space. Such models serve as a basis for in silico screening of cultivation conditions and genetic modification constructs, supporting technological decision-making and scaling processes from laboratory to industrial levels. Inclusion of biotechnological applications into the contour of systemic modeling of complex biological systems appears to be an important direction for further development of interdisciplinary research at the intersection of mathematics, biology, and engineering sciences.

## Conflict of interest

The authors declare no conflict of interest.
